# Production of IgG1-based bispecific antibody without extra cysteine residue via intein-mediated protein trans-splicing

**DOI:** 10.1038/s41598-021-98855-3

**Published:** 2021-09-30

**Authors:** Hiroki Akiba, Tomoko Ise, Satoshi Nagata, Haruhiko Kamada, Hiroaki Ohno, Kouhei Tsumoto

**Affiliations:** 1 Center for Drug Design Research, National Institutes of Biomedical Innovation, Health and Nutrition, Ibaraki, Osaka 567-0085 Japan; 2 Graduate School of Pharmaceutical Sciences, Kyoto University, Sakyo-ku, Kyoto, 606-8501 Japan; 3 School of Engineering, The University of Tokyo, Bunkyo-ku, Tokyo, 133-8656 Japan; 4 The Institute of Medical Science, The University of Tokyo, Minato-ku, Tokyo, 108-8639 Japan

**Keywords:** Proteins, Antibody therapy, Proteins

## Abstract

A major class of bispecific antibodies (BsAbs) utilizes heterodimeric Fc to produce the native immunoglobulin G (IgG) structure. Because appropriate pairing of heavy and light chains is required, the design of BsAbs produced through recombination or reassembly of two separately-expressed antigen-binding fragments is advantageous. One such method uses intein-mediated protein trans-splicing (IMPTS) to produce an IgG1-based structure. An extra Cys residue is incorporated as a consensus sequence for IMPTS in successful examples, but this may lead to potential destabilization or disturbance of the assay system. In this study, we designed a BsAb linked by IMPTS, without the extra Cys residue. A BsAb binding to both TNFR2 and CD30 was successfully produced. Cleaved side product formation was inevitable, but it was minimized under the optimized conditions. The fine-tuned design is suitable for the production of IgG-like BsAb with high symmetry between the two antigen-binding fragments that is advantageous for screening BsAbs.

## Introduction

Bispecific antibodies (BsAbs) are widely used as therapeutic agents and have been successful for example as T cell engagers^1,2^. Various methods have been developed for their production. BsAbs are divided into two major classes: low-molecular-weight BsAbs without the Fc part of immunoglobulin G (IgG) proteins, such as diabodies and peptide-linked single-chain variable fragments; and BsAbs bearing the Fc part of IgG so that the fundamental characteristics of the Ig proteins are maintained. In the latter class of BsAbs with two different antigen-binding fragments (Fabs), the Fc part is often engineered into heterodimeric structures. Several designs are available as heterodimeric Fc^3–10^. A major disadvantage of BsAbs based on heterodimeric Fc is the mispairing of heavy and light chains during production. In the so-called “light chain problem”, this mispairing occurs when two different variable regions are expressed in the same cell^1^. Because the heavy and light chain dimer formation is independent of the complementarity determining region, the theoretical yield of the properly paired BsAb is only 25%. To overcome this problem, various techniques have been developed^1^. For example, use of common light chains diminishes this problem^11^. In another approach, two constant regions inside the Fab part, on the heavy and light chains each, are interchanged^12^. Several mutants to distinguish between the two pairs of the constant regions are also available^13–15^.

**Figure 1:**
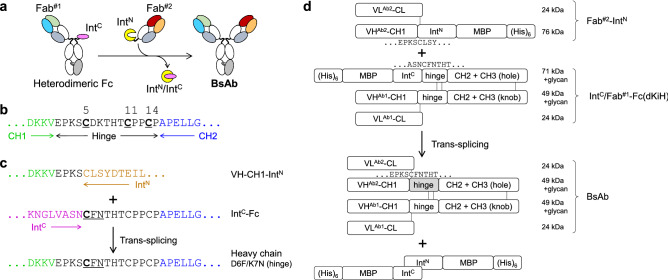
Design of polypeptides for intein-mediated protein trans-splicing (IMPTS). (**a**) General concept. (**b**) Sequence of the native hinge sequence of human IgG1. Cys residues (underlined) are numbered by the positions inside the hinge. (**c**) Designed IMPTS reaction to produce a native-like hinge sequence. Cys-Phe-Asn (underlined) mutated from the original Cys-Asp-Lys in the spliced product is optimal for IMPTS. The underlined Cys acts as the catalytic extein residue. (**d**) Polypeptide chains used in IMPTS and the products. Grey-colored hinge in the BsAb product contains two amino acid mutations.

When two Fabs are expressed separately, the problem diminishes^5–7^. A systematic approach to accomplish this is the use of post-translational conjugation or recombination of the polypeptides bearing the two Fab parts^16–20^. These methods are not always suitable for large-scale production, but are beneficial to screen for and optimize the combination of the variable regions in designing BsAbs. One such technology is intein-mediated protein trans-splicing (IMPTS)^21^. In IMPTS, N- and C-terminal fragments of intein (Int^N^ and Int^C^) are each fused to two different polypeptides and when the two components are mixed under reduced condition, spontaneous reaction occurs to form a peptide bond between the two polypeptides. Compared to other tags to enable recombination^16,19,22,23^, IMPTS is advantageous in that the third component, an enzyme, is not required, and minimal substrate peptide is left on the product because the Int^N^-Int^C^ complex is released^21^. Various applications take advantage of these features of IMPTS^20,24–30^; BsAbs with the IgG1 structure without the light chain problem have also been developed accordingly (Fig. 1a)^17,18,31^.

IMPTS is a reaction mediated by nucleophilic attack of the side chain of Cys residue at the N-terminus of C-extein (or the + 1 position to the C-terminus of Int^C^)^21^. Naturally occurring DNA polymerase III (DnaE) intein polypeptides (Int^N^ and Int^C^) from *Nostoc punctiforme* PCC73102 (Npu) have been reported to have high activity^32,33^. For the efficient IMPTS activity to occur for Npu DnaE, consensus amino acid residues surrounding the Cys + 1 of C-extein have been well characterized. It was reported that C-extein residues starting with CXN (X: various, preferentially Phe) are crucial^33–35^, whereas N-extein residues ending with AEY (or the − 3 to − 1 positions of the Int^N^ N-terminus) are preferred, but not essential^32,36^. Major side reactions to reduce the reaction yield of IMPTS are known as N-cleavage and C-cleavage, which are hydrolytic reactions mediated by reacting nucleophiles at the N-terminus of Int^N^ and the C-terminus of Int^C^, respectively^37,38^.

For using IMPTS in IgG1-like BsAb production^17,18,31^, the Int^N^/Int^C^ pair is incorporated in the hinge region (Fig. 1a). In previous reports, the consensus sequence of IMPTS on the extein (CFN) was newly incorporated in the flexible part of the hinge to achieve this design, and thus, the three amino acids were left in the hinge of the final BsAbs^18,31^. This additional Cys residue can disturb ordinary disulfide pairing, or may form a disulfide bond with an external molecule. These phenomena may reduce the homogeneity of the produced BsAb molecules, and may disturb the assay system throughout the selection of BsAbs. In this study, we designed C-extein using one of the natural Cys residues of the hinge to enable a normal-like hinge structure for the BsAb, and optimized the design for better expression and purification.

## Results and discussion

### Design of intein-fused antibody fragments

We used one of the Cys residues naturally available in the hinge region of human IgG1 as the reaction center at the N-terminus of C-extein (Fig. 1b). Because the dihedral angles formed around the Pro residue are completely different from other canonical amino acids, we avoided modifying the Pro-rich region in the hinge. Instead, Cys at the 5th position of the hinge was used as the residue for the reaction. When we used a natural IgG1 hinge sequence as the extein, the reaction did not occur, as expected from the preference of extein sequence reported^33–35^. Then, the surrounding residues were manipulated from (CH1)…EPKSC…(Int^N^) and (Int^C^)…*C*DKTHT…(hinge-Fc) in the original design to (CH1)…EPKSC…(Int^N^) and (Int^C^)…*CFN*THT…(hinge-Fc) (italics, reacting extein Cys and mutation) to optimize the C-extein. Using these polypeptides, the modified hinge (CH1)…EPKSCFNTHT…(hinge-Fc) without extra amino acids or extra Cys was produced in an IMPTS reaction (Fig. 1c). With the expectation of an efficient IMPTS reaction, we employed Cfa^39^, a high-efficiency intein mutant of Npu DnaE. To increase the productivity, maltose binding protein (MBP) tags were fused at the N-terminus of Int^C^ and at the C-terminus of Int^N^ in addition to the hexahistidine tag [(His)_6_] for affinity purification. In a previous report, CD40 extracellular domains were fused to the N-terminus of Int^C^^17,31^. Similar strategy was employed in this study but avoided use of a protein tag in the same family as the targets of the antibodies (TNFR2 and CD30). For heterodimerization of the Fc part, a well-characterized disulfide-bonded knobs-into-holes (dKiH) technique^3^ was applied using the CH3-domain allotype G1m, as described previously^40^. One half-heavy chain (HC) carried (His)_6_-MBP-Int^C^-Fc with “hole” mutations of dKiH, trans-spliced with the other half-HC VH-CH1-Int^N^-MBP-(His)_6_ to produce a full-HC containing the “hole” mutations. The other full-HC carried “knob” mutations of dKiH. The complete design of the polypeptides used for BsAb formation is described in Fig. 1d. The protein sequence is described in the Supporting Information.

The above design was applied for producing BsAbs targeting TNFR2 and CD30 as a model case. The VH region of an antibody against TNFR2, viz., TR109, was fused with CH1-Int^N^-MBP-(His)_6_, and the plasmid encoding this protein was co-transfected with the light chain (LC) to yield Fab^TNFR2^-Int^N^. Further, “knob” mutations of dKiH were introduced into the HC of an antibody against CD30, viz., T104^41,42^. The plasmid encoding this knob-mutated T104 HC was co-transfected with the LC and (His)_6_-MBP-Int^C^-Fc (with hole mutations) in a tripartite manner to yield Int^C^/Fab^CD30^-Fc. Both protein assemblies were expressed in Expi293F cells in 30–300 mL culture, and were separated from the expression medium by immobilized metal-affinity chromatography, followed by purification via size-exclusion chromatography (SEC) (Supporting Figs. S1 and S2). From 300-mL cultures, 100 nmol (14 mg) Int^C^/Fab^CD30^-Fc and 30 nmol (3 mg) Fab^TNFR2^-Int^N^ were obtained.

Intein sequences composing Fab^#2^-Int^N^ were compared between Cfa and Npu in this purification strategy. Cfa intein was advantageous over Npu intein, with ca. threefold higher production yield for the Int^N^-fused chain (1.3 mg versus 0.49 mg per 100 mL culture) after SEC (Fig. S3). Higher chemical stability of this mutant likely contributed to this result^39^. In addition, Int^N^-fused chain without MBP tag was compared with MBP-fused Fab^#2^-Int^N^. The production increased by twice (0.92 mg versus 2.0 mg per 30 mL culture; equivalent to 40% increase in mole from 15 to 21 nmol) and the purity was increased (70% versus 85% analyzed in SDS-PAGE) by MBP fusion (Fig. S4). When we first made attempt for IMPTS using natural hinge sequence as extein, tag-free and MBP-tagged constructs were compared in an overnight reaction at 37 °C in the presence of 2 mM dithiothreitol (DTT) (Fig. S5). As the result, complicated pattern of bands was observed without MBP tag in addition to similar level of C-cleaved side product in the two conditions, and made analysis difficult. Based on these results, we used Cfa intein fused with MBP tag (Fig. 1d) for the downstream optimization and analysis.

### Identification of the IMPTS product

**Figure 2:**
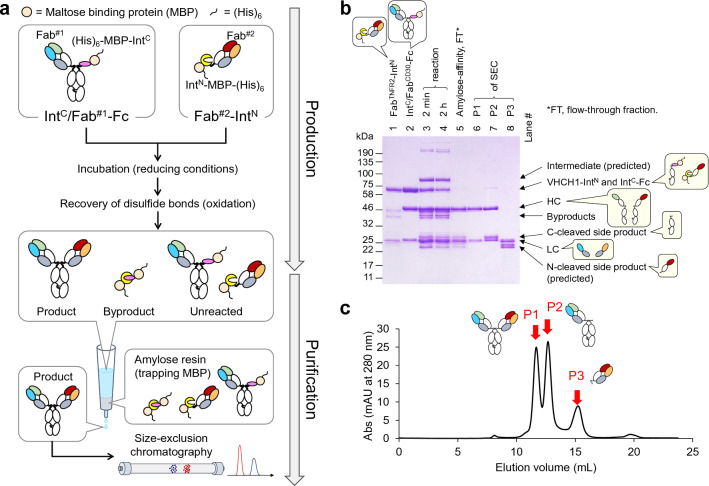
**(a)** Production and purification of bispecific antibody in this study. (**b)** SDS-PAGE analysis of the proteins throughout the production and purification process. The band corresponding to each polypeptide chain is indicated with arrows. (**c)** Size-exclusion chromatogram of the flow-through fractions of the amylose-affinity separation at initial conditions (6 h incubation at 37 °C with 2 mM DTT). The peaks analyzed in (**b**) are indicated with red arrows.

Schematic representation of the protocol of the IMPTS reaction and purification is presented in Fig. 2a. A reducing agent is required for initiating the IMPTS reaction. For the initial tests, excess Fab^TNFR2^-Int^N^ (15 μM) was mixed with the other protein (Int^C^/Fab^CD30^-Fc; 10 μM) and 2 mM DTT, and incubated at 37 °C for 6 h. The reaction was monitored in SDS-PAGE (Fig. 2b, lanes 1–4). After re-oxidation of the disulfide bonds using oxidized glutathione, MBP-fused protein fragments were removed from the reaction mixture using MBP-specific amylose resins. In the SDS-PAGE analysis, bands corresponding to HC and LC were observed (Fig. 2b, lane 5). Bands with the molecular weights of N- and C-cleaved side products were also observed. SEC for further purification of the flow-through component of affinity separation yielded three peaks (P1, P2, P3 of Fig. 2c). In the SDS-PAGE analysis, P1 was indicated as the BsAb main product (Fig. 2b, lane 6). P2 contained 25-kDa polypeptide which was estimated as a C-cleaved side product (lane 7). For P3, HC was missing and additional 23-kDa band was present (Fig. 2b, lane 8), and the presence of an N-cleaved side product was estimated.

**Figure 3:**
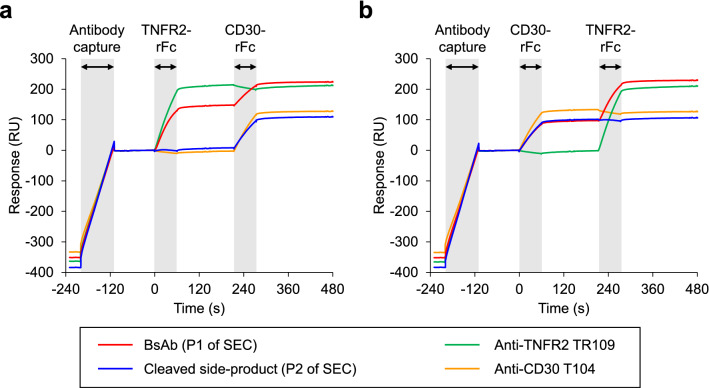
Interaction of the antibodies captured on a sensor chip with recombinant antigen proteins analyzed by surface plasmon resonance. (**a**) TNFR2-rFc and CD30-rFc as the 1st and 2nd analytes, respectively. (**b**) CD30-rFc and TNFR2-rFc as the 1st and 2nd analytes, respectively.

Binding analysis was performed to confirm the main and side products in P1 and P2. Sequential binding of recombinant TNFR2 and CD30 extracellular domains, each fused with rabbit IgG1 Fc (TNFR2-rFc and CD30-rFc, respectively), was analyzed by surface plasmon resonance (SPR). Human chimeric monoclonal antibodies (mAbs) of the original antibodies were used as a reference. mAbs or BsAbs were captured on a sensor chip using anti-human Fc antibody, and recombinant antigen molecules interacted as analytes. In the first experiment, TNFR2-rFc and CD30-rFc interacted as follows (Fig. 3a): anti-TNFR2 TR109 interacted only with TNFR2-rFc (the 1st analyte), and anti-CD30 T104 interacted only with CD30-rFc (the 2nd analyte), as expected. For the two products of IMPTS, contents of P1 interacted with both analytes, whereas contents of P2 interacted only with CD30-rFc. The same results were obtained when the order of the analytes was reversed (Fig. 3b). Because C-cleavage results in the production of monovalent Fab^CD30^-Fc, this observation supported our prediction that P1 contained BsAb main product and P2 contained a C-cleaved side product of the IMPTS reaction.

Presence of the expected IMPTS product BsAb in P1 was also demonstrated by mass spectrometry. The upper band of the SDS-PAGE for the BsAb (lane 6, Fig. 2b) was digested with a Trypsin/Lys-C mixture, and analyzed. Peptides from both the HCs, i.e., Fab^TNFR2^-Fc(hole) and Fab^CD30^-Fc(knob), were detected (Figs. S6 and S7). A hinge peptide of Fab^TNFR2^-Fc(hole) containing the catalytic extein residue was also observed, which supported the design of the proposed reaction mechanism (Fig. S8).

### Optimization of the IMPTS reaction

C-cleaved side product (yield of 26% to the precursor Int^C^), observed as the 2nd peak in the SEC, was produced more than the BsAb main product (15%). This undesirable side product had to be minimized. We first scanned the reaction time, and 2 h was found to be sufficient (Fig. S9); the subsequent experiments were conducted for 2 h duration in 250-μL reactions.

**Figure 4:**
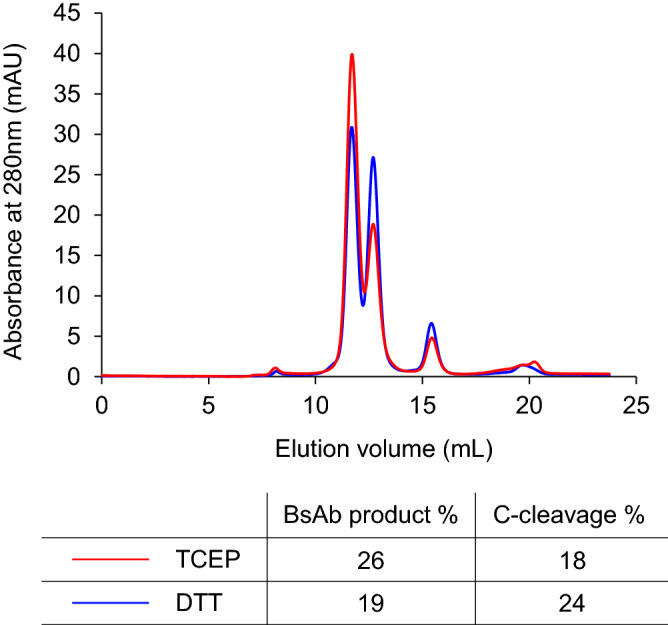
Comparison of the effect of DTT (blue) and TCEP (red) as the reducing agent via size-exclusion chromatography. Values of % yield of the BsAb product and C-cleaved side product were determined as the ratio of the products to the precursor Int^C^/Fab^#1^-Fc in mol. The quantity of the product was calculated from the area under the chromatogram.

In a major mechanism, unwanted cleavage of intein is considered to be initiated by the nucleophilic attack of external thiols, such as of DTT, on the thioester bond formed between Int^N^ and N-extein in the splicing reaction (N-cleavage); succinimide formation in Int^C^ to release C-extein may follow (C-cleavage)^37^. In a previous report, a cleaved side product was observed when the trans-splicing rate, dependent on the extein structure, was relatively low^38^. In this case, high concentrations of DTT inhibited trans-splicing and promoted thiol-mediated cleavage, and substitution of the reducing agent from thiol to phosphine was effective^38^. On the basis of this, we used tris(2-carboxyethyl)phosphine (TCEP) instead of DTT. The BsAb was successfully produced (25% of the precursor) over the cleaved side product (18%) when 2 mM TCEP was used, but the yield of the side product diminished only slightly (Fig. 4). Higher yield of the BsAb using TCEP suggests partial thiol-dependent cleavage^37^. Conversely, the yield of BsAb decreased when the concentration of the reducing agents was lower (Figs. S10 and S11). A higher concentration of DTT or TCEP was necessary, which indicated that reduction of the disulfide bond associated with extein Cys was the rate-determining step. Because a comparable amount of cleaved product was observed when either DTT or TCEP was used, it is inferred that a thiol-independent mechanism dominated the production of the side product found as the 13-mL peak in SEC (Fig. 2c).

Fab^TNFR2^-Int^N^ (15 μM) was 50% excess to Int^C^/Fab^CD30^-Fc (10 μM) at the initial condition. When the concentration of Fab^TNFR2^-Int^N^ was reduced to 10 μM, and the ratio was 1:1, the yield did not change largely from 25 to 23% (Fig. S12); thus, the excess Fab^TNFR2^-Int^N^ was not necessary. Further, decreasing the temperature to 25 °C reduced the yield to 16% (Fig. S12), indicating that a higher temperature was favorable. The yield did not increase when the IMPTS reaction was conducted for 24 h using TCEP (Fig. S13), which is consistent with the result obtained using DTT (Fig. S9). Based on the above experiments, the optimized conditions were: Int^C^/Fab^#1^-Fc, 10 μM; Fab^#2^-Int^N^, 10–15 μM; TCEP, 2 mM; and incubation for 2 h or more, at 37 °C. When the yield is high enough, as at the optimized condition, SEC enables the separation of BsAb from the side product. We propose that this optimized method is sufficient for BsAb screening systems, but the use of size-exclusion chromatography is not suitable for large scale production. Further optimization is required for development into manufacturing process.

Prolonged incubation in the presence of a reducing agent may potentially accelerate shuffling of HC and LC. To evaluate this potential unwanted reaction, a similar experiment was conducted as described previously^18^. First, Int^C^/Fab^#1^-Fc and Fab^#2^-Int^N^ with interchanged heavy and light chains were produced; Int^C^/Fab^#1^-Fc contained the light chain of TR109 and the HC of T104, whereas Fab^#2^-Int^N^ contained the light chain of T104 and the HC of TR109 (Fig. S14). These fragments do not interact with CD30 or TNFR2 unless shuffling occurs. Second, the IMPTS reaction was conducted for 24 h at 37 °C, under reducing conditions using 2 mM TCEP as the harshest condition, as applied earlier. Interaction of the produced L/H-interchanged BsAb with CD30 or TNFR2 was analyzed using SPR (Figs. S15 and S16). No interaction was observed even when a 20-fold higher concentration of the antibody was used. Hence, this condition does not promote shuffling.

We next compared the design of N- and C-exteins with the previously reported ones^18,31^. In one design by Han et al., consensus C-extein sequence CFN is inserted, so that 3 additional amino acids are present in the product BsAb^31^. In another design by Hofmann et al., consensus N- and C-extein sequences AEY and CFN are inserted, and 6 amino acids are present in the product BsAb^18^. IMPTS reaction was conducted in the above optimized conditions. As the result, there was dramatical differences among the three designs compared (Fig. S17). A low level of spliced product (5%) was obtained by CFN insertion, and the cleaved side product was dominant. On the other hand, AEYCFN insertion resulted in higher yield of the spliced product (32%), and C-cleavage was minimized. Our strategy to maintain the number of Cys residues in the hinge region (FN mutation) resulted in intermediate yield (25%). Design of extein in the present study offers a competitive choice under the strategy to maintain the number of Cys in hinge. Local sequences at around junction between intein and extein is the determinant of the reaction efficiency as known for protein splicing^32–36,43^, but both N- and C-cleaved side products were present at more than negligible level in all the three reactions. It has been reported that large extein protein could promote cleavage side reactions^38^, and the use of large extein (Fab and Fc) in combination with large MBP on the other side of intein might be disadvantageous.

### Analysis of bispecific interaction

**Figure 5:**
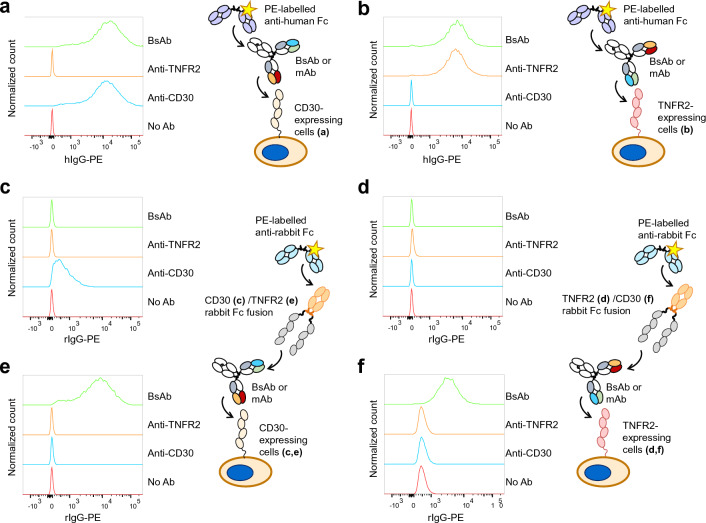
Cellular systems to analyze bispecific binding monitored using flow cytometry. Right shift of the population indicates binding. **a,b)** Interaction of the mAbs and the BsAb with the cells expressing CD30 **(a)** and TNFR2 **(b)**. (**c–f**) Crosslinking of the antigen-expressing cells and recombinant antigen protein using the mAbs and BsAb, analyzed using recombinant TNFR2 and CD30 fused to rabbit Fc (rFc). (**c**) CD30-RB and CD30-rFc, (**d**) TNFR2-RB and TNFR2-rFc, (**e**) CD30-RB and TNFR2-rFc, (**f**) TNFR2-RB and CD30-rFc.

Antigen-expressing cells were used to acquire evidence of bispecific binding of the CD30/TNFR2 BsAb. First, the interaction of mAbs and the BsAb with the cells was analyzed using a flow cytometer. Anti-CD30 T104 and BsAb were found to bind with CD30-expressing cells (CD30-RB), whereas anti-TNFR2 TR109 and BsAb bound to TNFR2-expressing cells (TNFR2-RB) (Fig. 5a,b). These results were consistent with the observations in the SPR analysis. Concentration dependency of the interaction indicated that the interaction of BsAb was weaker than that of mAbs to the cells expressing the respective antigens (Figs. S18 and S19), due to the absence of binding avidity. Next, the recombinant antigen was allowed to further interact with the antibody-bound cells. T104 was capable of binding simultaneously to CD30-RB and CD30-rFc (Fig. 5c). Both TR109 and BsAb were silent to this activity, and bivalent binding was required. Similarly, either T104 or BsAb did not bind to TNFR2-RB and TNFR2-rFc simultaneously, while TR109 showed marginal binding activity (Fig. 5d). Conversely, BsAb bridged CD30-RB and TNFR2-rFc (Fig. 5e), as well as TNFR2-RB and CD30-rFc (Fig. 5f). This result provides direct evidence of bispecific binding. A weak association between TNFR2-RB and CD30-rFc was observed (Fig. 5f), likely due to the expression of CD153 (or CD30 ligand) on Ramos cells^44^, that interact with recombinant CD30-rFc^45^, although BsAb-mediated interaction was still evident.

In addition to bispecific binding, Fc-mediated responses such as antibody-dependent cellular cytotoxicity are important functions of IgG-like BsAbs. Effect of two-amino acids mutation on these responses is unknown. Although we propose the present method to be developed as an effective system for screening IgG-like BsAbs in terms of the antigen-binding functions, further studies are required if this method is developed for manufacturing therapeutic or diagnostic BsAbs.

## Conclusion

We successfully produced an IgG1-like BsAb without extra Cys by IMPTS, using native Cys in the hinge region as the catalytic part. Even under optimized conditions, a significant amount of the cleaved side product was observed, because a thiol-independent mechanism was present. The yield of main and side products was comparable to previously reported design with additional Cys. The produced BsAbs are almost homogeneous, with high symmetry between the two HCs. Thus, the method would serve for screening BsAbs with biological activities dependent on the macromolecular structure of the antibody molecule. Such a systematic approach for BsAb screening is now under investigation.

## Methods

### Expression vectors for chimeric and BsAbs

DNA encoding the variable regions of the heavy and light chains were cloned into pFUSE-CHIg-hG1 and pFUSE2-CLIg-hK (InvivoGen), respectively, for the production of monoclonal antibodies T104 and TR109. The knob-chain of dKiH was produced by introducing mutations in the pFUSE-CHIg-hG1 vector using the PrimeSTAR Mutagenesis Basal Kit (Takara). Int^C^-fused hole-chain of dKiH and Int^N^ genes were synthesized by Genewiz, and cloned into pFUSE-CHIg-hG1 to substitute the original polypeptide-encoding nucleotides. The gene encoding MBP was cloned from pMAL-p5x (New England Biolabs), and cloned into the aforementioned vectors. Variable regions were cloned into these vectors using the NEBuilder HiFi DNA Assembly Kit (New England Biolabs).

### Expression of monoclonal antibodies

Recombinant chimeric antibodies were produced using Expi293 Expression System (Thermo Fisher Scientific) following the manufacturer’s instructions. Briefly, Expi293F cells were transfected with the vectors encoding the heavy and light chains (30 μg each) per cell culture of 60 mL using the ExpiFectamine 293 Transfection Kit (Thermo Fisher Scientific). The cell culture was incubated on a shaker for 6–7 days post transfection, and the cells were removed by centrifugation at 6,000 × *g* for 20 min. The culture supernatant was filtered through a 0.20 μm filter, and the antibodies were captured in a 1 mL HiTrap Protein A HP Column (Cytiva). The column was washed with 20 mL phosphate-buffered saline (PBS), and the antibodies were subsequently eluted with 5 mL citrate buffer (100 mM, pH 3.0). The eluate was dialyzed in PBS and used without further purification.

### Expression and purification of Int^C^/Fab^CD30^-Fc and Fab^TNFR2^-IntN

Proteins were produced using the Expi293 Expression System in the same way as chimeric mAbs. Int^C^/Fab^CD30^-Fc and Fab^TNFR2^-Int^N^ were produced in a 300-mL scale expression medium by co-transfection of two and three plasmid vectors of equivalent mass. Proteins were purified as described previously^40^. Briefly, after 6–7 days of culture, the supernatant was dialyzed into buffer A (20 mM Tris–HCl, 300 mM NaCl, pH 7.5), and the proteins were captured on four 1 mL columns of cOmplete His-Tag Purification Resin (Roche Diagnostics). The column was washed with buffer A containing 20 mM imidazole, and the proteins were eluted with buffer A containing 500 mM imidazole. The eluate was dialyzed using buffer A containing 1 mM EDTA, and was subjected to final purification using a HiLoad Superdex200 26/600 or 16/600 column (Cytiva) in the same buffer. Chromatograms were recorded using AKTA pure 25 (Cytiva) with a flow cell path length of 2 mm. Molecular weights indicated for SEC chromatogram are recorded using Gel Filtration Calibration Kits (LMW and HMW, Cytiva).

### Production of BsAbs

Schematic representation of BsAb production has been provided in Fig. 2a. Int^C^/Fab^CD30^-Fc and Fab^TNFR2^-Int^N^ were mixed at final concentrations of 10 μM and 15 μM, respectively, in a 250-μL or 300-μL solution of buffer A containing 1 mM EDTA and the reducing agent (DTT or TCEP at variable concentrations). The solution was incubated under variable conditions and then dialyzed using buffer A containing 1 mM EDTA, for 4 h. The solution was further dialyzed overnight into buffer A containing 1 mM oxidized glutathione. MBP-fused side products and unreacted fragments were removed using a 0.5 mL Amylose resin column (New England Biolabs). The column was washed with 2 mL buffer A, and the flow-through and wash solution were combined and were concentrated by an ultrafiltration unit (Amicon Ultra-4 30 K, Merck-Millipore) to 200 μL. The final purification of the product was conducted in a Superdex 200 Increase 10/300 GL column (Cytiva) in the same buffer. For subsequent analysis, the buffer was replaced with PBS during ultrafiltration using Amicon Ultra-4 (30 K, Merck-Millipore).

### SPR

Interaction of the mAbs and BsAbs with the recombinant proteins was monitored using a Biacore T200 instrument (Cytiva). The measurements were performed in PBS supplemented with 0.05% Tween 20 (pH 7.4), at a flow rate of 30 μL/min at 25 °C. The antibodies were captured on a CM5 chip immobilized with anti-human IgG Fc using Human Antibody Capture Kit (Cytiva) following the manufacturer’s instructions. The antibodies (1 μg/mL) were run for 120 s to capture ca. 400 RU. As antigens, the extracellular domains of human TNFR2 and CD30 were subcloned into a pcDNA3-based rabbit IgG Fc-fusion vector, as described previously^46^. The Fc-fusion proteins were expressed and purified as the chimeric antibodies described above. The antigen proteins (10 nM) were run for a contact time of 60 s, and dissociation times of 60 s and 180 s for the 1st and 2nd contacts, respectively.

### Mass spectrometry

Peptides were extracted from Coumassie brilliant blue-stained SDS-PAGE gels. In-gel reduction and alkylation were performed as described^47^. Digestion was performed using the Rapid Digestion-Trypsin/Lys-C Kit (Promega), for 60 min at 70 °C. The peptides were withdrawn from the gel by the addition of 1% trifluoroacetic acid, and then desalted using an OMIX C18 chip (Agilent). LC–MS/MS analysis was performed as described^48^, with the modification in the LC program consisting of a linear gradient of the buffers A/B from 95/5 to 55/45 in 30 min (buffer A, 0.1% formic acid and 2% acetonitrile; buffer B, 0.1% formic acid and 90% acetonitrile). Data analysis was conducted using Proteome Discoverer 1.4 (Thermo Fisher Scientific). Peptides were identified by the SEQUEST algorithm, using the library consisting of the starting materials and the product of the IMPTS reaction. Deviation of up to 5 ppm for the precursor ion, and up to 0.01 Da for the fragment mass was allowed.

### Flow cytometry

The full length coding sequence of TNFR2 was gene-synthesized, and inserted between the BamHI and XhoI sites of the pcDNA6 myc His A vector (Thermo Fisher Scientific). The constructed vector was transfected into the Ramos-Blue cell line (InvivoGen) using an electroporator (Amaxa). After selection with blasticidin, several rounds of cell sorting and cell cloning by the limiting dilution method were carried out to establish TNFR2-transfected Ramos Blue cells. The cells were incubated with the mAbs or the BsAb in PBS containing 5% fetal bovine serum and 0.1% sodium azide, for 1 h on ice, and washed twice with the same buffer. To analyze the binding of the antibodies, Fcγ fragment-specific anti-human IgG labeled with R-Phycoerythrin (R-PE, #109–116-170, Jackson Immunoresearch Laboratories) was used. To analyze the crosslinking of cells and the recombinant antigen protein, cells were incubated with the mAbs or the BsAb (10 μg/mL), washed twice, and then incubated with CD30-rFc or TNFR2-rFc (50 ng/mL) for 1 h on ice, and the binding of rFc was probed with anti-rabbit IgG labeled with R-PE (#111–005-046, Jackson Immunoresearch Laboratories). The cells were separated and analyzed using a BD LSRFortessa X-20 Flow Cytometer (BD Biosciences). Data were analyzed in FlowJo v10.7.1 (BD Biosciences).

## Supplementary Information

Supplementary Information.
