# Supplementary material for: Production of IgG1-based bispecific antibody without extra cysteine residue via intein-mediated protein trans-splicing
Source: Sci Rep. 2021 Sep 30;11:19411. doi: 10.1038/s41598-021-98855-3 (PMC8484483; doi:10.1038/s41598-021-98855-3)
Supplement: Supplementary file 1 — Supplementary Information. [file 41598_2021_98855_MOESM1_ESM.pdf]

Supplementary Information for:

Production of IgG1-based bispecific antibody without extra cysteine residue via intein-mediated protein trans-splicing

Hiroki Akiba[1,2*], Tomoko Ise[1], Satoshi Nagata[1], Haruhiko Kamada[1], Hiroaki Ohno[2], and Kouhei Tsumoto[1,3,4*]

[1]Center for Drug Design Research, National Institutes of Biomedical Innovation, Health and Nutrition, Ibaraki, Osaka 567-0085, Japan

[2]Graduate School of Pharmaceutical Sciences, Kyoto University, Sakyo-ku, Kyoto 606-8501, Japan

[3]School of Engineering, The University of Tokyo, Bunkyo-ku, Tokyo 133-8656, Japan

[4]The Institute of Medical Science, The University of Tokyo, Minato-ku, Tokyo 108-8639, Japan

Sequences of the heavy chain constant region for intein-mediated protein trans-splicing

$(His)_6$-MBP-Int^C-hinge(partial)-Fc(hole)

MVRLPLQCVLWGCLLTAVHPSGHHHHHHGGSGKIEEGKLVIWINGDKGYNGLAEVGKKFEKDTGIKV
TVEHPDKLEEKFPQVAATGDGPDIIFWAHDRFGGYAQSGLLAEITPDKAFQDKLYPFTWDAVRYNGK
LIAYPIAVEALSLIYNKDLLPNPPKTWEEIPALDKELKAKGKSALMFNLQEPYFTWPLIAADGGYAF
KYENGKYDIKDVGVDNAGAKAGLTFLVDLIKNKHMNADTDYSIAEAAFNKGETAMTINGPWAWSNID
TSKVNYGVTVLPTFKGQPSKPFVGVLSAGINAASPNKELAKEFLENYLLTDEGLEAVNKDKPLGAVA
LKSYEEELVKDPRIAATMENAQKGEIMPNIPQMSAFWYAVRTAVINAASGRQTVDEALKDAQGSGSS
GGVKIISRKSLGTQNVYDIGVEKDHNFLLKNGLVASNCFNTHTCPPCPAPELLGGPSVFLFPPKPKD
TLMISRTPEVTCVVVDVSHEDPEVKFNWYVDGVEVHNAKTKPREEQYNSTYRVVSVLTVLHQDWLNG
KEYKCKVSNKALPAPIEKTISKAKGQPREPQVCTLPPSRDELTKNQVSLSCAVKGFYPSDIAVEWES
NGQPENNYKTTPPVLDSDGSFFLVSKLTVDKSRWQQGNVFSCSVMHEALHNHYTQKSLSLSPGK

CH1-hinge(partial)-Int^N-MBP-$(His)_6$

(VH)-ASTKGPSVFPLAPSSKSTSGGTAALGCLVKDYFPEPVTVSWNSGALTSGVHTFPAVLQSSGL
YSLSSVVTVPSSSLGTQTYICNVNHKPSNTKVDKKVEPKSCLSYDTEILTVEYGFLPIGKIVEERIE
CTVYTVDKNGFVYTQPIAQWHNRGEQEVFEYCLEDGSIIRATKDHKFMTTDGQMLPIDEIFERGLDL
KQVDGLPGGSGGGGSGGSKIEEGKLVIWINGDKGYNGLAEVGKKFEKDTGIKVTVEHPDKLEEKFPQ
VAATGDGPDIIFWAHDRFGGYAQSGLLAEITPDKAFQDKLYPFTWDAVRYNGKLIAYPIAVEALSLI
YNKDLLPNPPKTWEEIPALDKELKAKGKSALMFNLQEPYFTWPLIAADGGYAFKYENGKYDIKDVGV
DNAGAKAGLTFLVDLIKNKHMNADTDYSIAEAAFNKGETAMTINGPWAWSNIDTSKVNYGVTVLPTF
KGQPSKPFVGVLSAGINAASPNKELAKEFLENYLLTDEGLEAVNKDKPLGAVALKSYEEELVKDPRI
AATMENAQKGEIMPNIPQMSAFWYAVRTAVINAASGRQTVDEALKDAQGHHHHHH

CH1-hinge-Fc(knob)

(VH)-ASTKGPSVFPLAPSSKSTSGGTAALGCLVKDYFPEPVTVSWNSGALTSGVHTFPAVLQSSGL
YSLSSVVTVPSSSLGTQTYICNVNHKPSNTKVDKKVEPKSCDKTHTCPPCPAPELLGGPSVFLFPPK
PKDTLMISRTPEVTCVVVDVSHEDPEVKFNWYVDGVEVHNAKTKPREEQYNSTYRVVSVLTVLHQDW
LNGKEYKCKVSNKALPAPIEKTISKAKGQPREPQVYTLPPCRDELTKNQVSLWCLVKGFYPSDIAVE
WESNGQPENNYKTTPPVLDSDGSFFLYSKLTVDKSRWQQGNVFSCSVMHEALHNHYTQKSLSLSPGK

Color represents each protein component.

Highlighted, reacting extein Cys; underlined, mutations introduced in the constant region of human IgG1.

Supplementary Figures

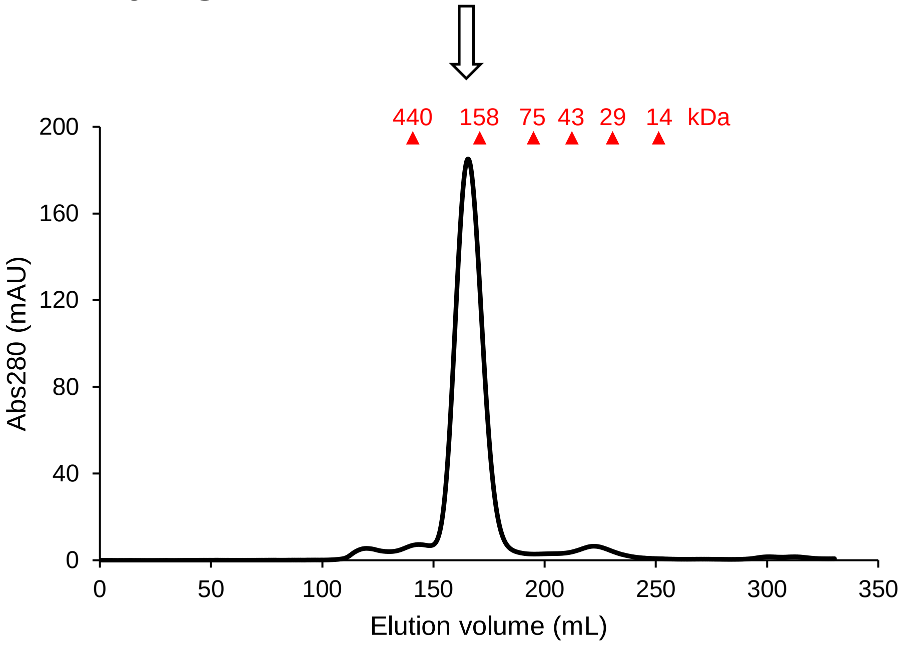

Figure S1. Size-exclusion chromatogram of Int^{C}/Fab^{CD30}-Fc (indicated with an arrow) obtained by transient expression in a 300-mL culture of Expi293F. Red triangles indicate the elution volume of size markers.

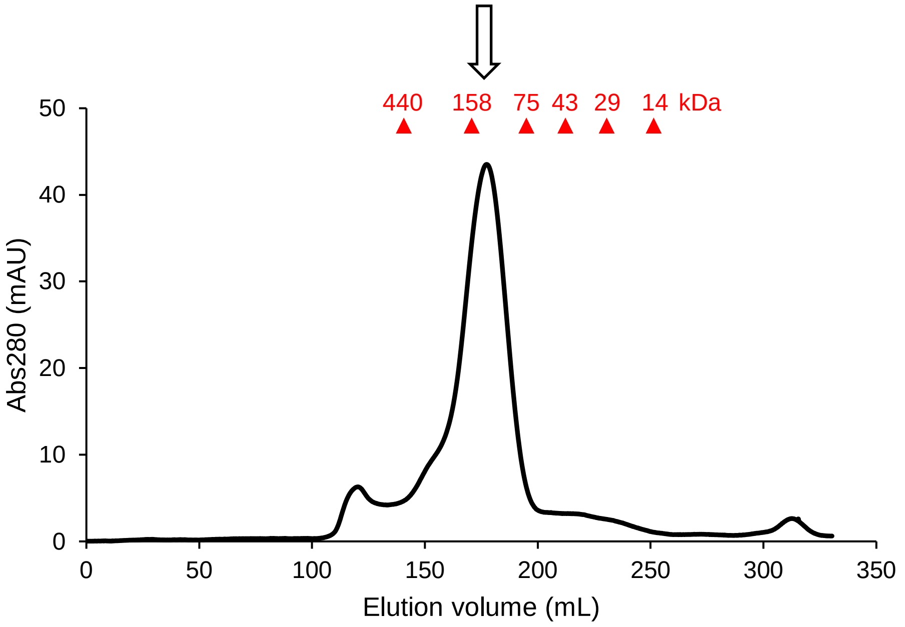

Figure S2. Size-exclusion chromatogram of Fab^{TNFR2}-Int^{N} (indicated with an arrow) obtained by transient expression in a 300-mL culture of Expi293F. Red triangles indicate the elution volume of size markers.

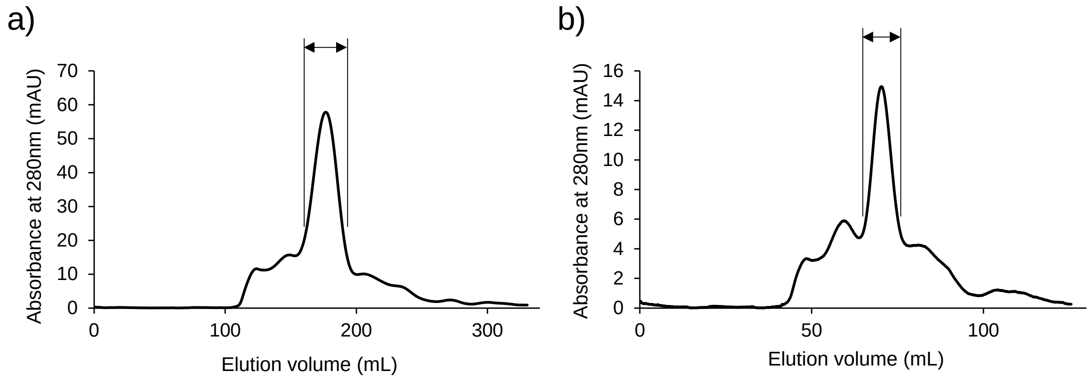

Figure S3. Size-exclusion chromatograms of Fab^{TNFR2}-Int^{N} using **a)** Cfa intein and **b)** Npu intein for the Int^{N} obtained by transient expression in 300-mL and 60-mL cultures of Expi293F, respectively, for **a** and **b**. Arrows indicate the peak area to calculate the yields.

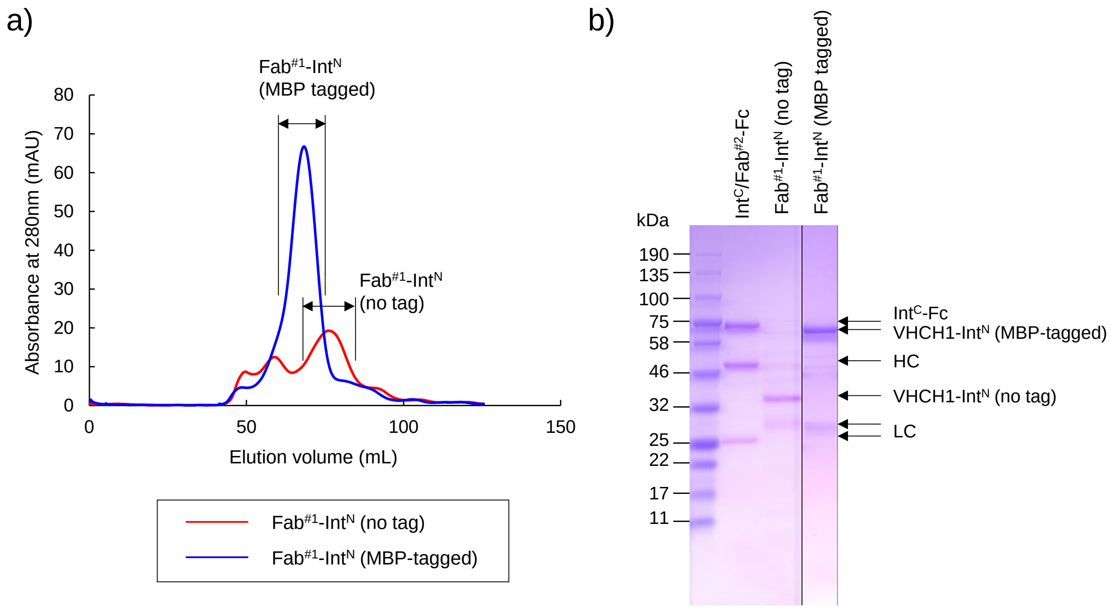

Figure S4. a) Size-exclusion chromatograms of Fab$^{\#1}$-Int^{N} designed without C-terminal tag (red) or fused with maltose binding protein (MBP) tag (blue), obtained by transient expression in 30-mL cultures of Expi293F. Arrows indicate the peak area to calculate the yields. **b)** SDS-PAGE analysis of the protein in the main peaks. Black line shows the boundary of the gel images from different gels.

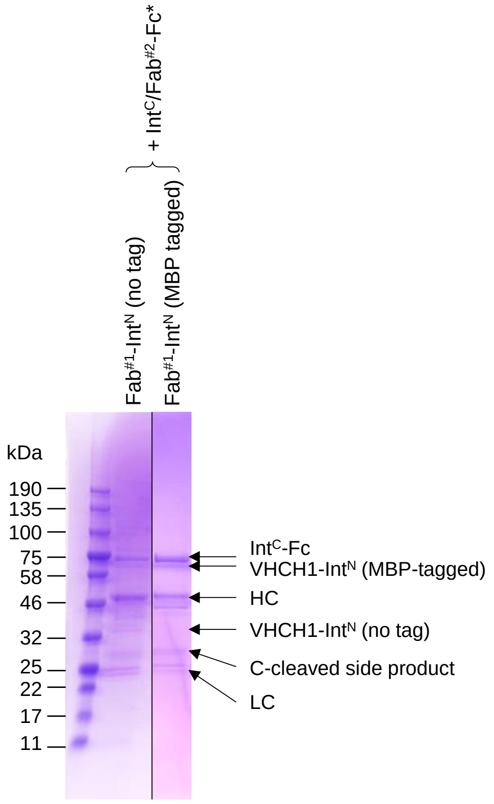

Figure S5. Comparison of the presence (MBP tagged) and absence (no tag) of C-terminal expression tag fused to IntN in an intein-mediated protein trans-splicing reaction using a Int^{C}-Fc construct using natural hinge sequence as the C-extein (with asterisk). Protein mixture after an overnight reaction at 37 °C in the presence of 2 mM dithiothreitol was analyzed in SDS-PAGE. Black line shows the boundary of the gel images from different gels.

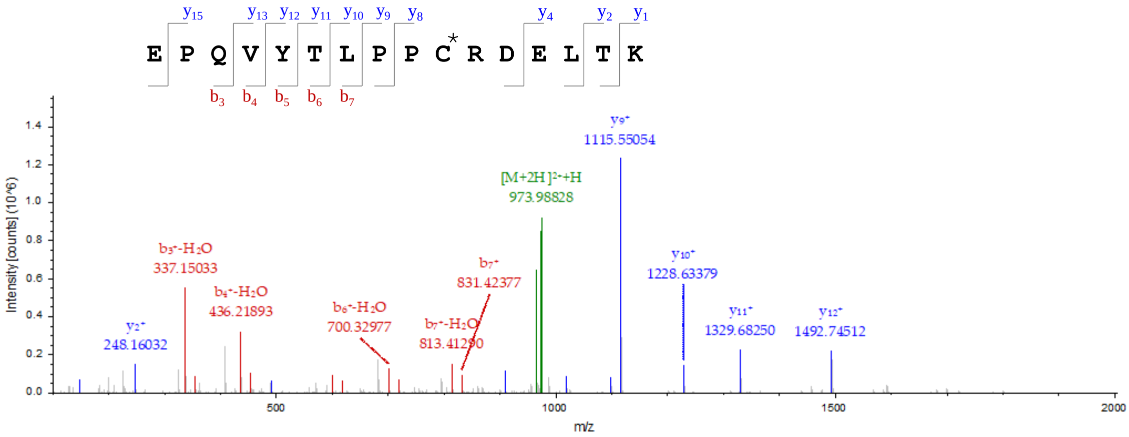

Figure S6. MS^{2} spectrum of the selected peptide originated from the mutated Fc of T104-Fc(knob). C*, carbamidomethylated cysteine.

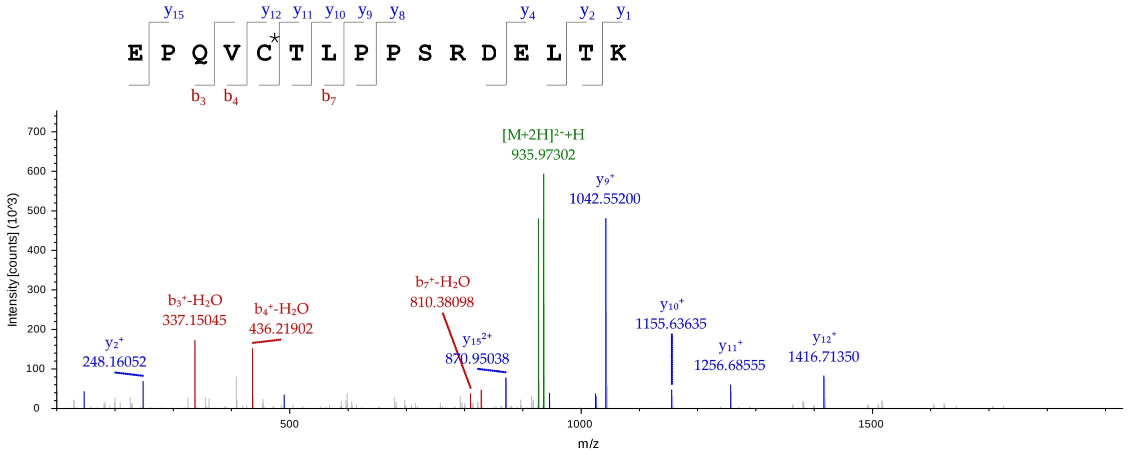

Figure S7. MS^2 spectrum of the selected peptide originated from the mutated Fc of TR109-Fc(hole). C*, carbamidomethylated cysteine.

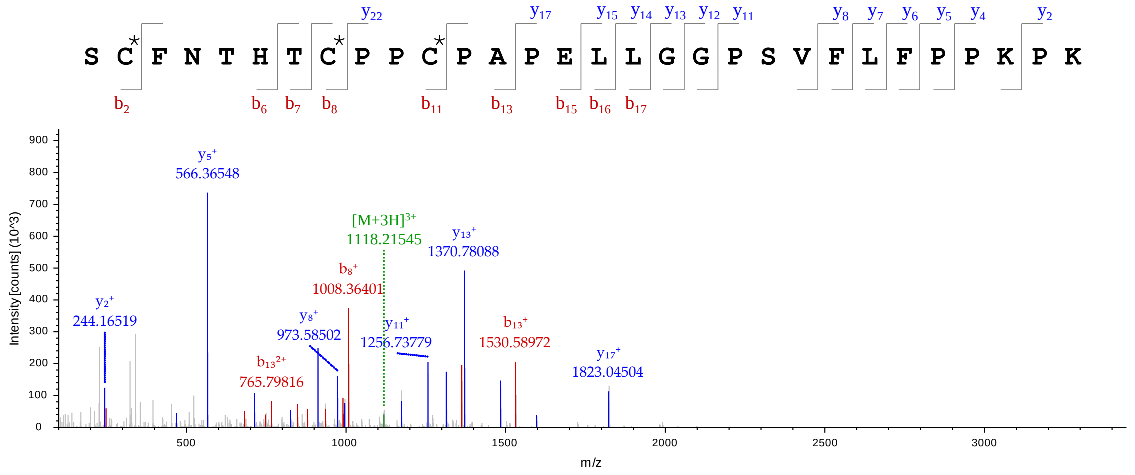

Figure S8. MS^2 spectrum of the peptide originated from the modified hinge region generated through the IMPTS reaction. C*, carbamidomethylated cysteine.

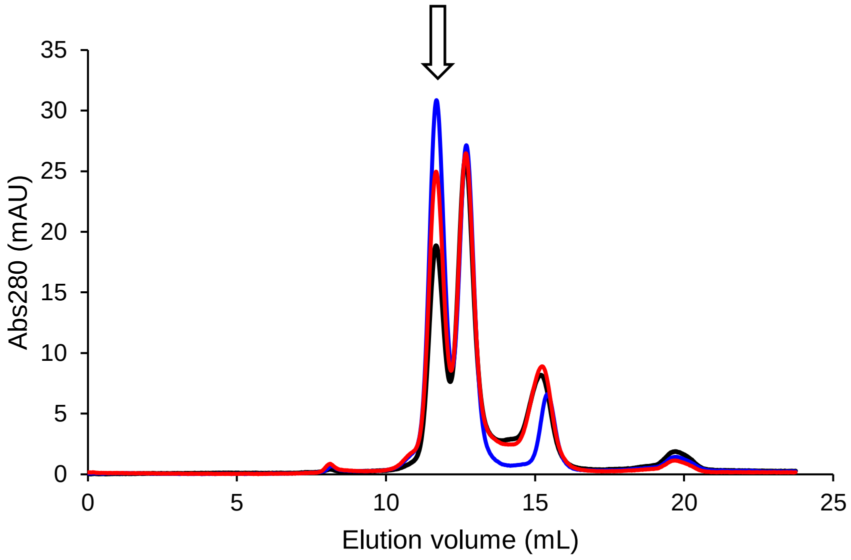

Reaction time	BsAb product %	C-cleavage %
6 h	15	26
2 h	19	24
0.5 h	12	25

Figure S9. Time course of the IMPTS reaction in the presence of DTT in the size-exclusion chromatogram of the flow-through fraction of the amylose-affinity separation. Black, 0.5 h; blue, 2 h; red, 6 h (original condition). The arrow indicates the BsAb product. Values of % yield of the BsAb product and C-cleaved side product were determined as the ratio of the products to the precursor Int^{C}/$Fab^{\#1}$-Fc in mol. The quantity of the product was calculated from the area under the chromatogram.

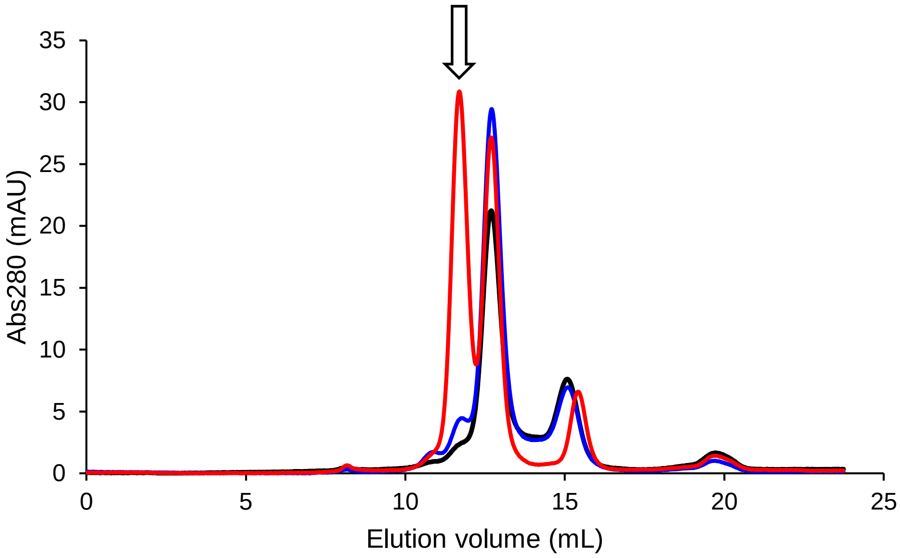

DTT concentration	BsAb product %	C-cleavage %
2 mM	19	24
0.5 mM	3	30
0.1 mM	2	23

Figure S10. Size-exclusion chromatograms of the flow-through fractions of the amylose-affinity separation in the conditions with lower concentrations of DTT. The concentration of DTT used was: black, 0.1 mM; blue, 0.5 mM; red, 2 mM (original condition). The arrow indicates the BsAb product. Values of % yield of the BsAb product and C-cleaved side product were determined as the ratio of the products to the precursor Int^{C}/$Fab^{\#1}$-Fc in mol. The quantity of the product was calculated from the area under the chromatogram.

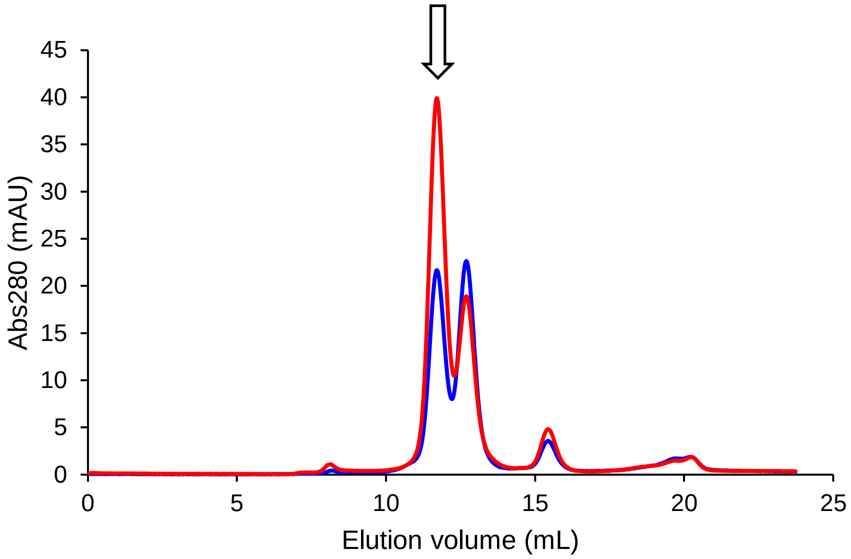

TCEP concentration	BsAb product %	C-cleavage %
2 mM (red line)	25	18
0.5 mM (blue line)	14	21

Figure S11. Size-exclusion chromatograms of the flow-through fractions of the amylose-affinity separation in the condition with lower concentration of TCEP. The concentration of TCEP used was: blue, 0.5 mM; red, 2 mM (original condition). The arrow indicates the BsAb product. Values of % yield of the BsAb product and C-cleaved side product were determined as the ratio of the products to the precursor $Int^{C}/Fab^{\#1}$-Fc in mol. The quantity of the product was calculated from the area under the chromatogram.

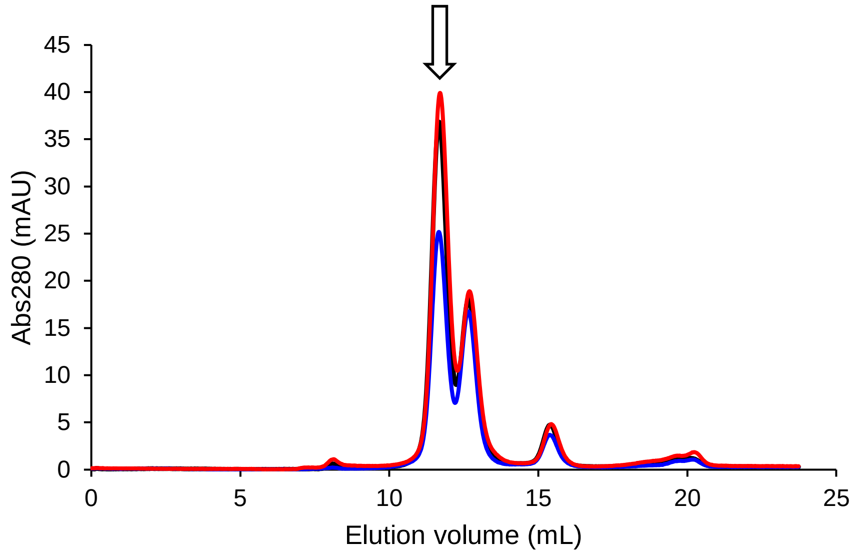

	BsAb product %	C-cleavage %
Int^C:Int^N = 1:1.5, 37 °C	25	18
Int^C:Int^N = 1:1, 37 °C	23	17
Int^C:Int^N = 1:1, 25 °C	16	15

Figure S12. Concentration-dependency of Fab^{TNFR2}-Int^N and the effect of the temperature in the size-exclusion chromatogram of the flow-through fraction of the amylose-affinity separation. Red, original condition (Int^C : Int^N = 1 : 1.5 at 37 °C); black, decreased amount of IntN fragment (Int^C : Int^N = 1 : 1 at 37 °C); blue, decreased temperature (Int^C : Int^N = 1 : 1 at 25 °C). The arrow indicates the BsAb product. Values of % yield of the BsAb product and C-cleaved side product were determined as the ratio of the products to the precursor Int^C/$Fab^{\#1}$-Fc in mol. The quantity of the product was calculated from the area under the chromatogram.

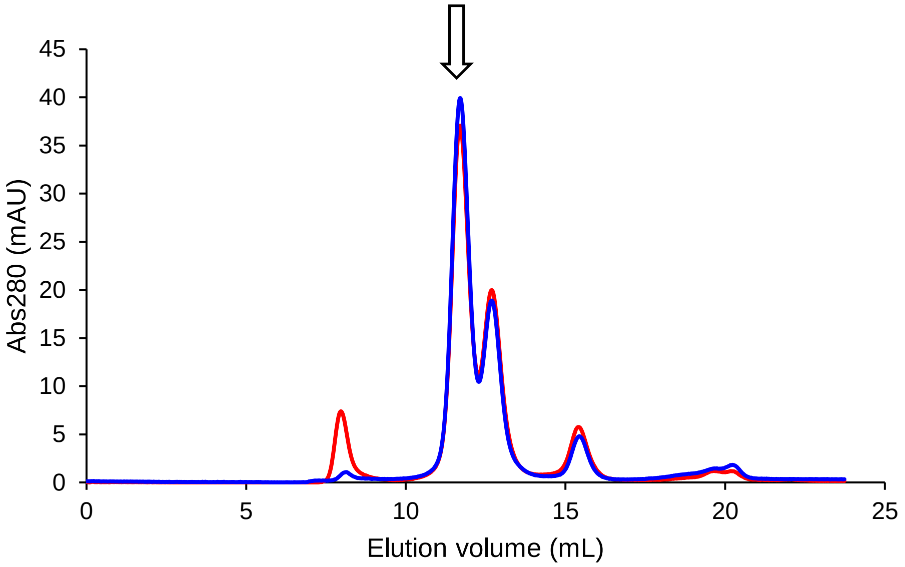

Figure S13. Time course of the IMPTS reaction in the presence of TCEP in the size-exclusion chromatogram of the flow-through fraction of the amylose-affinity separation. Blue, 2 h (original condition); red, 24 h. The arrow indicates the BsAb product.

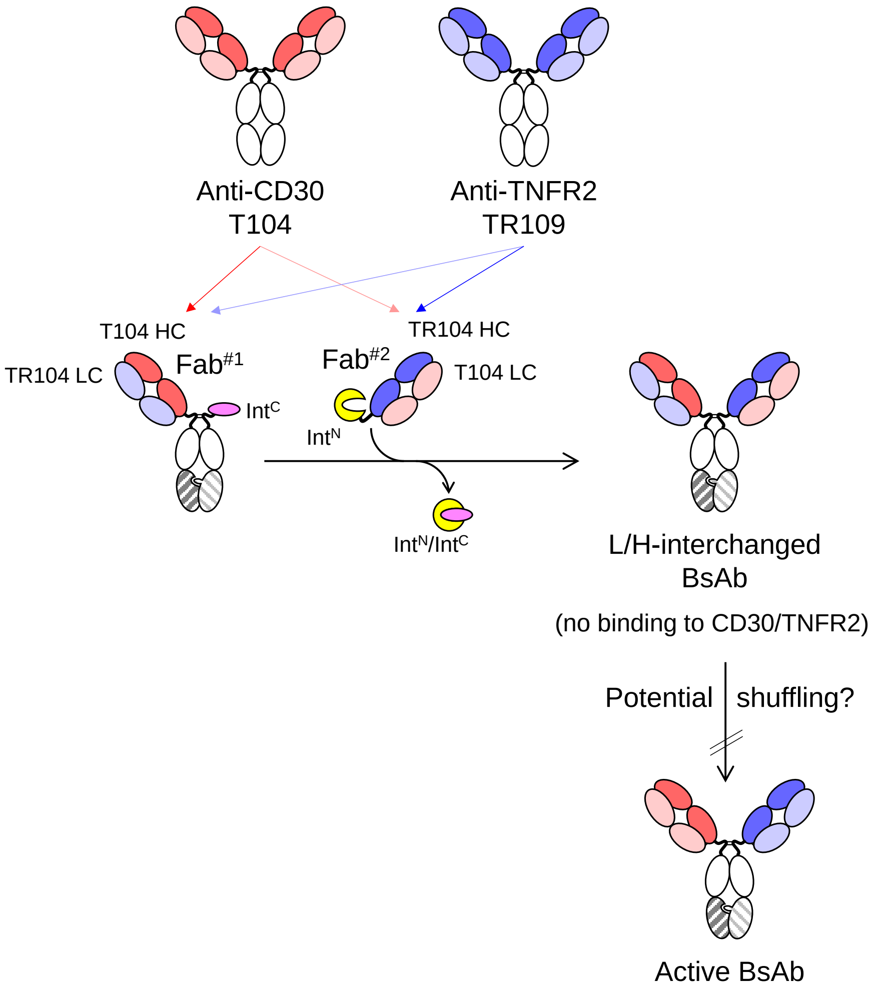

Figure S14. Design of the L/H-interchanged BsAb and the potential production of active BsAb through light- and heavy-chain shuffling (not observed in this study).

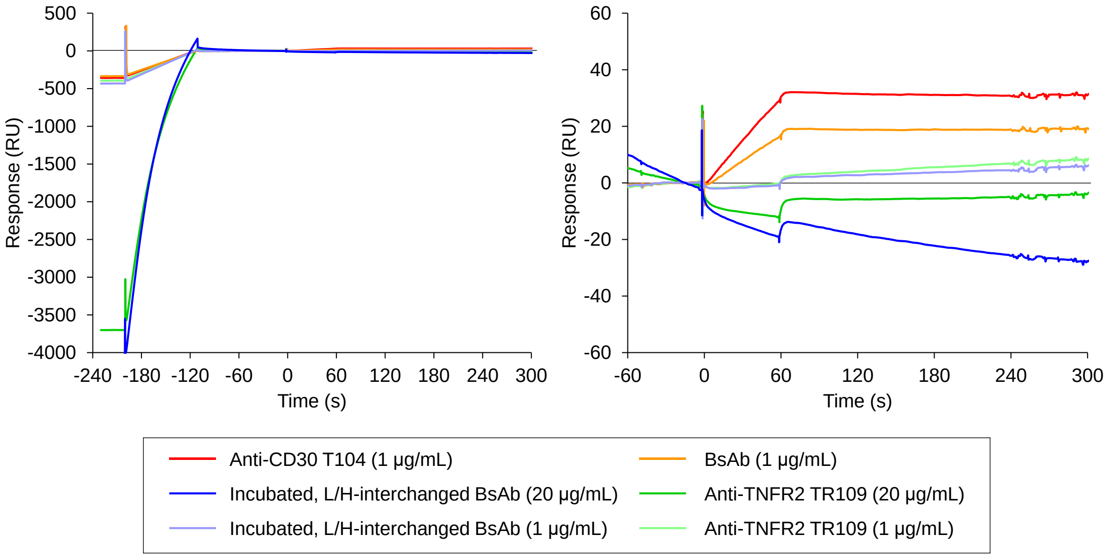

Figure S15. Interaction of the produced L/H-interchanged BsAb with recombinant CD30-rFc. Left, capture of the antibodies (−200 to −110 s), contact with CD30-rFc (0 to 60 s), and dissociation (60 to 300 s). Right, enlarged view of the contact and dissociation. Baseline did not settle during the waiting time when antibodies were captured at high concentration (20 µg/mL).

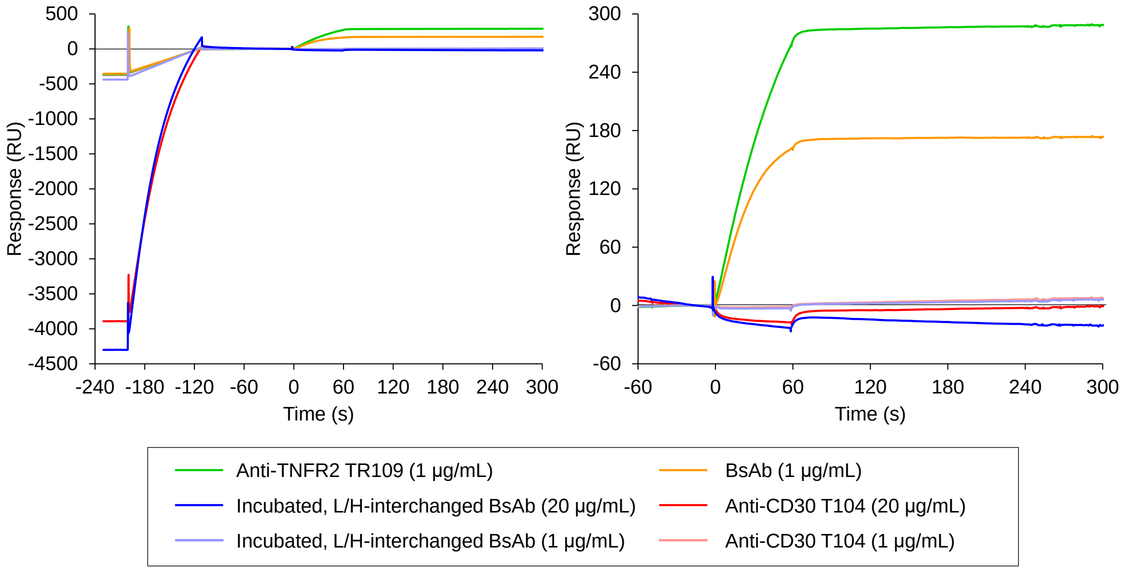

Figure S16. Interaction of the produced L/H-interchanged BsAb with recombinant TNFR2-rFc. Left, capture of the antibodies (−200 to −110 s), contact with TNFR2-rFc (0 to 60 s), and dissociation (60 to 300 s). Right, enlarged view of the contact and dissociation. Baseline did not settle during the waiting time when antibodies were captured at high concentration (20 µg/mL).

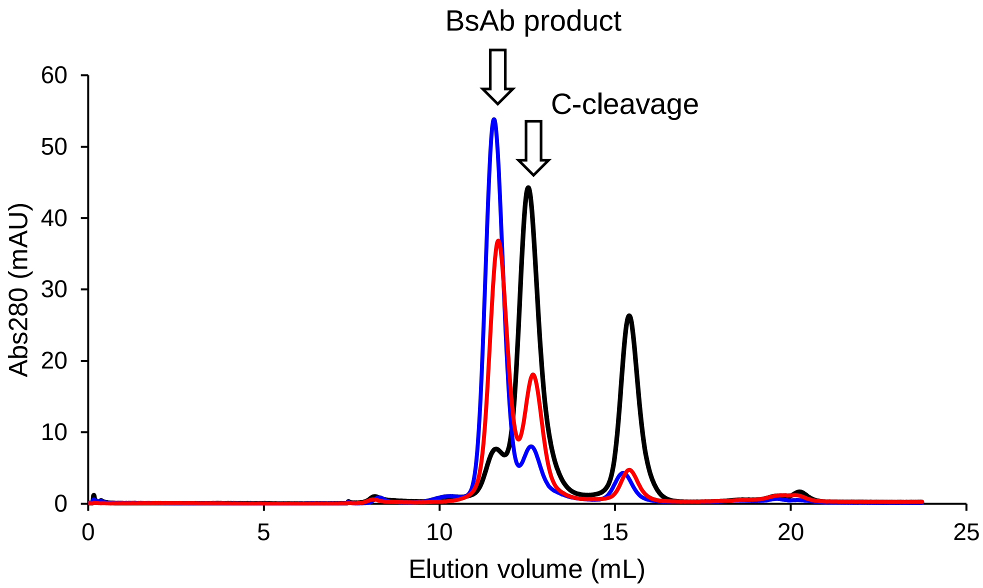

	BsAb product %	C-cleavage %
FN mutation (this study) (red line)	23	17
AEYCFN insertion (Hofmann et al) (blue line)	32	8
CFN insertion (Han et al) (black line)	5	47

Figure S17. Size-exclusion chromatograms of the flow-through fractions of the amylose-affinity separation after the IMPTS reaction as a comparison of three different designs of extein sequences in the hinge region. Values of % yield of the BsAb product and C-cleaved side product were determined as the ratio of the products to the precursor $Int^{C}/Fab^{\#1}$-Fc in mol. The quantity of the product was calculated from the area under the chromatogram.

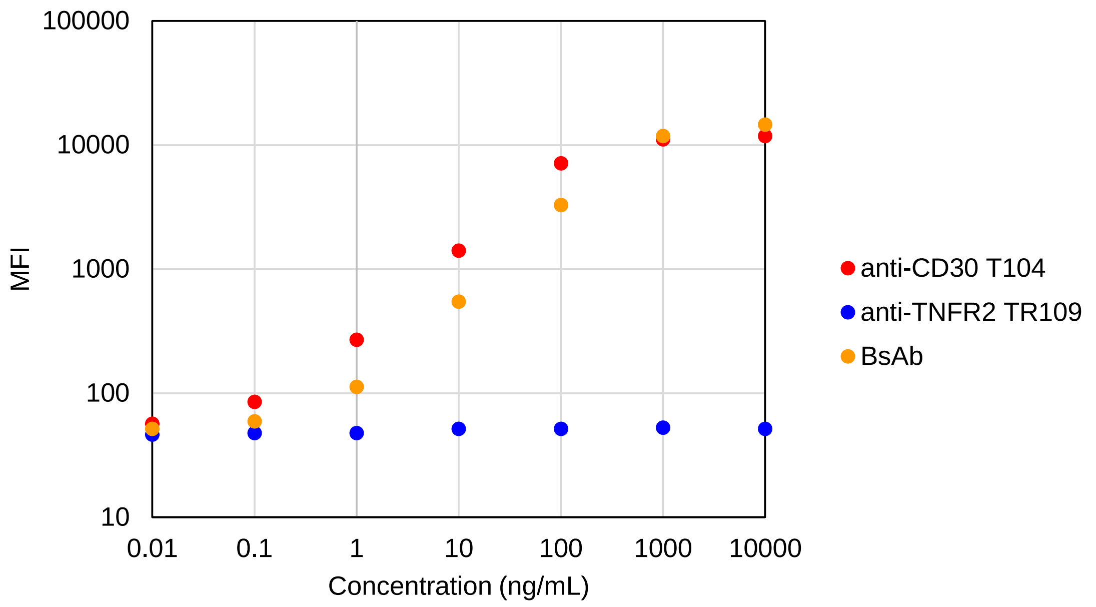

Figure S18. Concentration dependency of median fluorescence intensity of T104 and the BsAb interacting with the CD30-expressing cells.

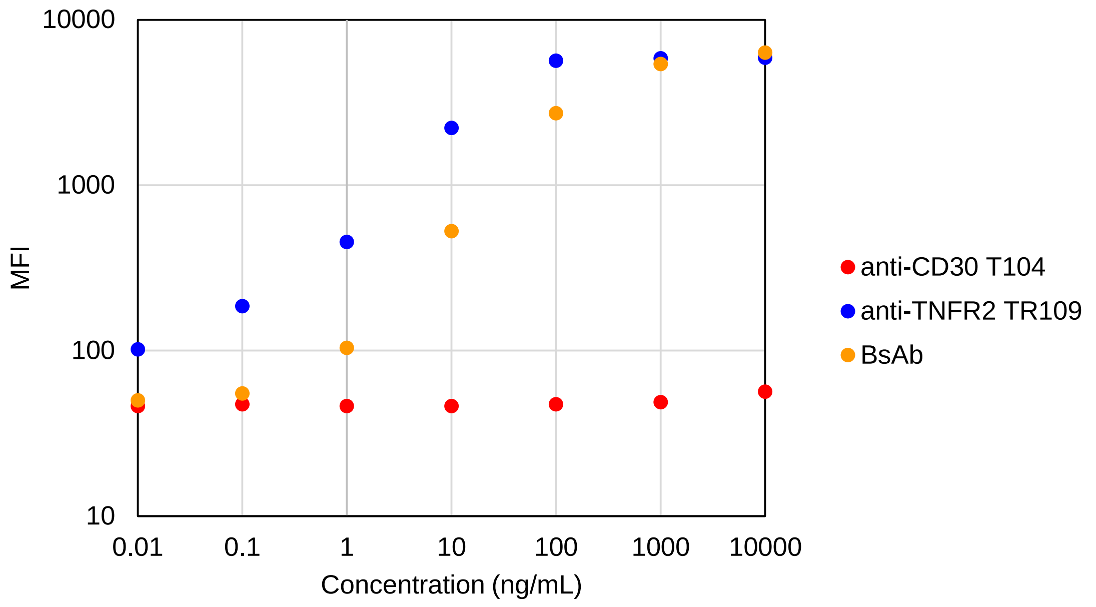

Figure S19. Concentration dependency of median fluorescence intensity of TR109 and the BsAb interacting with the TNFR2-expressing cells.
